# Mortality and repeated poisoning after self-discharge during treatment for acute poisoning by substances of abuse: a prospective observational cohort study

**DOI:** 10.1186/s12873-018-0219-9

**Published:** 2019-01-11

**Authors:** Odd Martin Vallersnes, Dag Jacobsen, Øivind Ekeberg, Mette Brekke

**Affiliations:** 1 Department of General Practice, University of Oslo, Oslo, Norway; 2 Department of Emergency General Practice, City of Oslo Health Agency, Oslo Accident and Emergency Outpatient Clinic, Oslo, Norway; 3 Department of Acute Medicine, Oslo University Hospital, Oslo, Norway; 4 Institute of Clinical Medicine, University of Oslo, Oslo, Norway; 5 Division of Mental Health and Addiction, Oslo University Hospital, Oslo, Norway; 6 Department of Behavioural Sciences in Medicine, University of Oslo, Oslo, Norway; 7 General Practice Research Unit (AFE), University of Oslo, Oslo, Norway

**Keywords:** Poisoning, Intoxication, Alcohol, Drug abuse, Self-discharge, Leaving against medical advice, Mortality, Repeated poisoning

## Abstract

**Background:**

Though substance use is a known risk factor for self-discharge, patients self-discharging during treatment for acute poisoning have not previously been described. We charted characteristics of patients self-discharging during treatment for acute poisoning by substances of abuse looking for associations between self-discharge, repeated poisoning, and death.

**Methods:**

All patients 12 years and older treated for acute poisoning by substances of abuse at an emergency outpatient clinic in Oslo, Norway, were included consecutively from October 2011 through September 2012. We collected data on gender, age, main toxic agent, suicidal intention, homelessness, history of severe mental illness, and self-discharge. Information on deaths was retrieved from the National Cause of Death Register. We did a multiple logistic regression analysis to look for associations between self-discharge and repeated poisoning and a Cox regression analysis for associations between self-discharge and death.

**Results:**

During one year, 1731 patients were treated for 2343 episodes of acute poisoning by substances of abuse. Two-hundred-and-sixty-six (15%) patients self-discharged during at least one poisoning episode. Self-discharging patients were older, median age 39 years vs 32 years (*p* <  0.001), more frequently homeless, 20/266 (8%) vs 63/1465 (4%) (*p* = 0.035), and the main toxic agent more frequently was an opioid, 82/266 (31%) vs 282/1465 (19%) (*p* <  0.001). Self-discharge was an independent risk factor for repeated poisoning. The adjusted odds ratio for two or more poisoning episodes during one year among self-dischargers was 3.0 (95% CI 2.2–4.1). The association was even stronger for three or more poisoning episodes, adjusted odds ratio 5.0 (3.3–7.5). In total, there were 34 deaths, 9/266 (3.4%) among self-discharging patients and 25/1465 (1.7%) among patients not self-discharging (*p* = 0.12). The adjusted hazard ratio for death among self-discharging patients was 1.6 (0.75–3.6).

**Conclusions:**

Self-discharge was associated with frequent poisonings by substances of abuse. Short-term mortality was doubled among self-discharging patients, though this increase was not statistically significant. Still, the increased risk of repeated poisoning marks self-discharging patients as a vulnerable group who might benefit from targeted post-discharge follow-up measures.

## Background

Self-discharging patients leave health facilities against medical advice or merely disappear. In general hospital populations, self-discharge rates of 1–3% have been reported [1–5]. Self-discharge gives reason for concern, as it is a risk factor both for readmissions and for excess mortality. In a study encompassing all unscheduled hospital admissions in a Canadian province during 20 years, discharge against medical advice tripled the risk of readmission within 30 days, and the risk of death at 90 days was increased by 150% [1]. Among nearly two million admissions to US veteran hospitals, the readmission rate following discharge against medical advice was 18% within 30 days, compared to 11% following regular discharge, while the risk of death at 60 days was increased by 10% [2]. In a state-wide register study of 270,000 trauma admissions in California, the 30-days readmission rate, regularly at 7 %, was increased to 17% following discharge against medical advice [3].

Self-discharge has been found to be more frequent among patients using alcohol or illicit drugs [2, 6]. A US nationwide register study found rates of discharge against medical advice in the range of 10–12% among patients treated for alcohol and substance related disorders compared to 1 % in general [4]. Among 34,000 patients brought by ambulance to an emergency department in a Canadian study, 55% of patients leaving without being seen had a substance abuse diagnosis, compared to 9 % among other patients [7]. A Korean study of 126,000 injury patients found that the risk of discharge against medical advice from the emergency department was nearly doubled when alcohol was involved [8]. In a UK study of patients treated for self-harm in general hospitals, the risk of self-discharge was increased by 49% among those using alcohol or illegal drugs [9].

Though patients using alcohol or illicit drugs have high rates of self-discharge, we have not found any studies describing patients self-discharging during treatment for substance use related poisoning, apart from the mere reporting of rates. Among patients treated for acute poisoning, the rates of self-discharge range from six to 11% in UK and Norwegian studies [10–13]. In a European multi-centre study of patients treated for recreational drug toxicity in emergency departments, the self-discharge rate was as high as 17% [14].

Acute poisoning is in itself a marker of increased risk. It is associated with excess long-term mortality, especially when related to substance use [15–17]. Repetition rates are also high. In previous Norwegian studies, as many as 30% of patients treated for acute poisoning presented with a new poisoning within a year [18], and 9 % of patients treated for acute poisoning by substances of abuse repeated within a week [19]. Hence, patients self-discharging during treatment for substance use related poisoning are an at-risk group in an at-risk situation, not previously described in detail.

### Aims

We charted characteristics of patients self-discharging during treatment for acute poisoning by substances of abuse. Furthermore, we looked for associations between self-discharge and short-term mortality, and between self-discharge and repeated poisoning.

## Methods

### Design

Prospective observational cohort study.

### Setting

The study was done at the Oslo Accident and Emergency Outpatient Clinic (OAEOC) in Oslo, Norway. The OAEOC is a primary care emergency outpatient clinic, serving the entire city at all hours. There are about 200,000 consultations a year. The majority of patients treated for acute poisoning by substances of abuse in Oslo, are treated at the OAEOC [20]. These patients are assessed according to a systematic observation procedure, and the median observation time is four hours [19]. Oslo is the capital city of Norway (population 613,285 as per 1 January 2012 [21]).

### Inclusion

We included all patients 12 years and older treated at the OAEOC for an acute poisoning by substances of abuse. Patients were included by the doctor treating them. Substances of abuse were defined as any potential substance of abuse including alcohol, prescription drugs, illegal drugs, and others. Patients were included consecutively during one year, from 1 October 2011 to 30 September 2012. Patients treated for multiple conditions were included if the poisoning in itself caused need for treatment or observation. Among 2733 eligible cases, 174 did not have a Norwegian national identity number and were excluded. In 216 cases the patient declined to participate. In the end, 2343 cases in 1731 patients were included.

### Data collection and classification

For all included cases, a registration form was completed by the doctor treating the patient. Any information missing in the form was collected, if available, from the electronic medical records. Information on deaths from 1 October 2011 to 31 December 2012 was retrieved from the National Cause of Death Register, using the patients’ unique Norwegian national identity number.

For each case, we registered age, gender, main toxic agent, homelessness, suicidal intention, previous history of severe mental illness, time of presentation, time of discharge, and whether the patient self-discharged during treatment. Diagnoses of main toxic agents were made by the doctor treating the patient. Main toxic agent was defined as the agent assessed to be most toxic among the agents taken, assumed doses considered. The diagnoses were based on all information available then and there. Subsequently, main toxic agents were grouped by us as ethanol, opioids, stimulants, gamma-hydroxybutyrate (GHB), benzodiazepines, and others. Suicidal intention was registered according to the assessment of the doctor treating the patient. Previous history of severe mental illness was assessed and registered by the doctor treating the patient based on information from local medical records, from the patient and/or the patient’s companions. Severe mental illness encompassed psychosis, bipolar disorder and severe personality disorders. Homelessness was defined as being registered without a permanent address in the National Registry, which was accessed via the electronic medical records. Self-discharge was defined as leaving without being seen by a doctor, disappearing during treatment or leaving against medical advice. For patients disappearing during treatment, time of discharge was defined as the time when they were registered as missing from the clinic in the electronic medical records.

By using the patient’s unique Norwegian national identity number, we could identify patients presenting more than once during the inclusion period. The collected information was collated for each patient. Main toxic agent for a patient with more than one presentation was defined as the main toxic agent most frequently diagnosed in that patient’s poisoning episodes. In case of even frequencies, we used the toxic agent we considered most serious, in the following order: opioids, stimulants, GHB, benzodiazepines, ethanol, others.

### Outcome measures

The main outcome measures were short-time mortality, defined as death during the inclusion period or the following three months; repeated poisoning once during the inclusion period; and repeated poisoning several times during the inclusion period. We looked for associations between self-discharge and these outcomes. Furthermore, we described characteristics of the self-discharging patients.

### Statistics

Statistical analyses were done in SPSS version 25 and in an online calculator from EpiTools epidemiological calculators [22]. Chi-square test was used when comparing proportions. Mann-Whitney U-test was used when comparing continuous variables. We did a Cox regression analysis to identify factors associated with short-term mortality, and multiple logistic regression analyses to identify factors associated with repeated poisoning.

### Ethics

The study was performed in accordance with the Helsinki declaration. It was approved by the Regional Committee South East for Medical and Health Research Ethics (REK nr 2010/1129–1) and the Oslo University Hospital Information Security and Privacy Office.

## Results

Among the 1731 patients included, 266/1731 (15%) self-discharged during at least one poisoning episode. In total, 324/2343 (14%) episodes ended with self-discharge. Self-discharging patients were older, median age 39 years vs 32 years (*p* <  0.001), more frequently homeless, 20/266 (8%) vs 63/1465 (4%) (*p* = 0.035), and the main toxic agent more frequently was an opioid, 82/266 (31%) vs 282/1465 (19%) (*p* <  0.001) (Table 1).

**Table 1:** Characteristics of self-discharging patients

	Self-dischargers *n* (%)	Non-self-dischargers *n* (%)	*p*-value
Males	184 (69)	952 (65)	0.21
Age^a^	39 (28–50)	32 (23–46)	< 0.001
Main toxic agent			
*Ethanol*	143 (54)	829 (57)	0.43
*Opioids*	82 (31)	282 (19)	< 0.001
*Stimulants*	12 (5)	93 (6)	0.31
*GHB*	11 (4)	74 (5)	0.63
*Benzodiazepines*	12 (5)	132 (9)	0.020
*Other*	6 (2)	55 (4)	0.30
Suicide attempt^b^	14 (5)	116 (8)	0.17
Severe mental illness	33 (12)	150 (10)	0.34
Homeless	20 (8)	63 (4)	0.035
Two or more poisonings^c^	89 (33)	186 (13)	< 0.001
Three or more poisonings^c^	56 (21)	62 (4)	< 0.001
Death^d^	9 (3.4)	25 (1.7)	0.12
Total	266 (100)	1465 (100)	

^a^Median (interquartile range)

^b^Suicidal intention in at least one poisoning episode

^c^During one year, i.e. during the inclusion period (1 October 2011–30 September 2012)

^d^Death during the inclusion period or the following three months (1 October 2011–31 December 2012)

*GHB* gamma-hydroxybutyrate

**Fig. 1:**
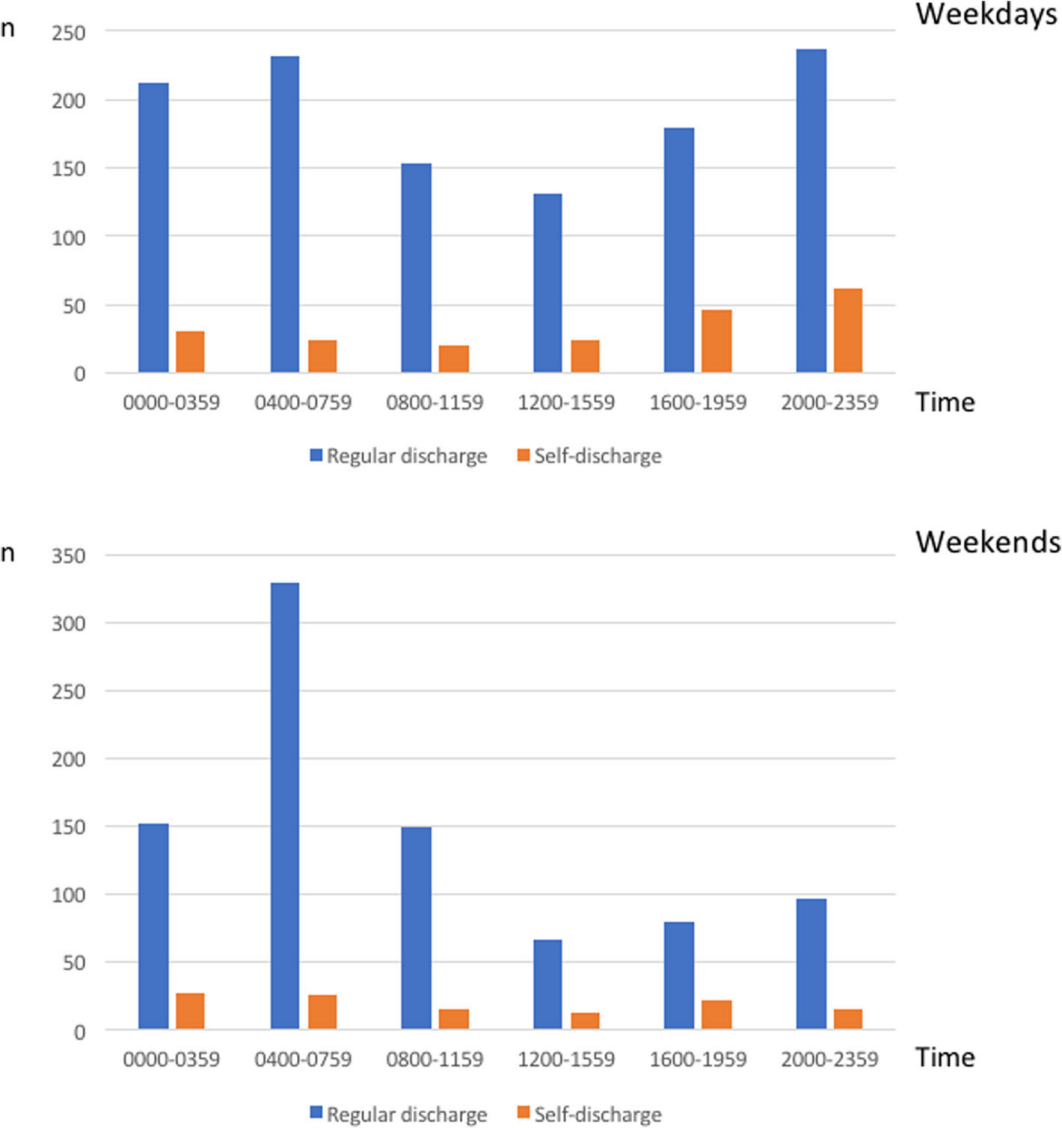
Time of discharge. Time of self-discharge was differently distributed than time of regular discharge, both on weekdays and on weekends (*p* < 0.001). Weekends include Saturdays, Sundays and public holidays

In cases ending with the patient self-discharging, the median length of stay was 2 h 45 min (interquartile range 1 h 35 min – 4 h 40 min), compared to 4 h 5 min (2 h 20 min – 5 h 25 min) when treatment at the OAEOC ended with medical discharge or sending the patient on to hospital (p <  0.001). Most self-discharges occurred on weekdays between 16:00 and midnight (Fig. 1).

Thirty-four patients died, 9/266 (3.4%) among self-discharging patients compared to 25/1465 (1.7%) among patients not self-discharging (*p* = 0.12) (Table 1). The hazard ratio for death among self-discharging patients in the univariate Cox regression analysis was 2.0 (95% confidence interval (CI) 0.92–4.2, *p* = 0.079), while the adjusted hazard ratio in the multivariate Cox regression analysis was 1.6 (95% CI 0.75–3.6, *p* = 0.21) (adjusted for age, gender, toxic agent, suicide attempt, severe mental illness, and homelessness).

Self-discharge was an independent risk factor for repeated poisoning, with an adjusted odds ratio of 3.0 (95% CI 2.2–4.1, *p* <  0.001) for presenting more than once during one year (Table 2). The association was even stronger for presenting more than twice, adjusted odds ratio 5.0 (95% CI 3.3–7.5, *p* <  0.001).

**Table 2:** Factors associated with two or more poisonings during one year – multiple logistic regression analysis

	Patients total	Patients with two or more poisonings	Crude			Adjusted		
	*n*	*n* (%)	Odds ratio	95% CI	*p*-value	Odds ratio	95% CI	*p*-value
Gender								
*Females*	595	72 (12)	1			1		
*Males*	1136	203 (18)	1.6	1.2–2.1	0.002	1.3	0.96–1.8	0.090
Age^a^			1.02	1.01 – 1.02	< 0.001	**1.02**	1.01–1.03	< 0.001
Main toxic agent								
*Ethanol*	972	106 (11)	1			1		
*Opioids*	364	121 (33)	4.1	3.0–5.5	< 0.001	**3.7**	2.7–5.1	< 0.001
*Stimulants*	105	22 (21)	2.2	1.3–3.6	0.003	**2.1**	1.2–3.6	0.008
*GHB*	85	11 (13)	1.2	0.63–2.4	0.57	1.4	0.70–2.8	0.34
*Benzodiazepines*	144	13 (9)	0.81	0.44–1.5	0.50	0.70	0.35–1.4	0.33
*Other*	61	2 (3)	0.28	0.07–1.2	0.077	0.27	0.06–1.1	0.075
Suicide attempt^b^	130	21 (16)	1.0	0.63–1.7	0.93	1.2	0.65–2.2	0.55
Severe mental illness	183	55 (30)	2.6	1.8–3.7	< 0.001	**2.9**	1.9–4.2	< 0.001
Homeless	83	27 (33)	2.7	1.7–4.4	< 0.001	1.3	0.78–2.3	0.29
Self-discharge^c^	266	89 (33)	3.5	2.6–4.7	< 0.001	**3.0**	2.2–4.1	< 0.001
Total	1731	275 (16)						

Adjusted odds ratios for significant associations are shown in bold types

^a^Continuous variable

^b^Suicidal intention in at least one poisoning episode

^c^Self-discharge in at least one poisoning episode

*CI* confidence interval, *GHB* gamma-hydroxybutyrate

## Discussion

### Summary of main findings

Self-discharge was associated with a three-fold increased risk of repeated poisoning, and a five-fold increased risk of repeating more than once. Short-time mortality was twice as high among self-discharging patients, though this increase was not statistically significant. Self-discharging patients were older, more frequently homeless, and the main toxic agent more frequently was an opioid.

### Self-discharge, repeated poisoning and death

We found self-discharge to be an independent risk factor for repeated poisoning by substances of abuse. The increased risk of repetition was high compared with previous findings of increased readmission rates following discharge against medical advice in general hospital populations [1–3].

The increased repetition rate among patients self-discharging during treatment for acute poisoning of abuse calls for concern, as risk of fatal overdose increases with increasing numbers of previous non-fatal overdoses [23–25]. In line with previous studies in general hospital populations [1, 2], we found an increased risk of death following self-discharge. However, the increase was not statistically significant in our study, probably a consequence of too small a sample for the short observation time.

The self-discharge rate of 15% among our patients was high compared to the rates of 1–3% reported in general hospital populations [1–5]. The rate was more comparable to the rate of 17% previously reported among patients treated for recreational drug toxicity [14], but higher than the rates of 6–11% reported among patients treated for acute poisoning in general [10–13]. Though higher rates of self-discharge have been reported among patients treated for self-harm, 23% among adolescents in a Korean study [26], and ranging from eight to 39% in a UK study encompassing 22 general hospitals [9], the rate of 15% marks our patient group as prone to self-discharge.

### Why do patients self-discharge?

There are clear indications that self-discharging patients are a troubled and vulnerable group. Garland et al. found readmission rates and mortality to be increased immediately following discharge against medical advice, then declining but still increased at least as long as six months later, leading them to speculate that the increases were related both to the acute condition and to characteristics of the patient [1]. In our study, self-discharging patients more often were opioid users, homeless, and of older age. In a Canadian study of hospitalised patients using illicit drugs, unstable employment, incarceration, daily heroin injection and younger age were associated with leaving against medical advice [27]. An Australian study of patients with deliberate self-poisoning found absconding patients more likely to have a mental illness and to be unemployed [28]. Among trauma patients in California, readmissions were more likely to be for psychiatric reasons following discharge against medical advice compared to regular discharge [3].

In a qualitative study, communication breakdown was found to be a major reason for self-discharge, encompassing health service providers using language that was hard to understand for patients, inconsistent messages from doctors and nurses, and sheer rudeness from health service providers [29]. Accordingly, clear and consistent communication in an easy to understand language along with good bedside manners, might partly remedy the situation. However, self-discharge is a complex phenomenon, associated with serious consequences. Self-discharging patients might benefit from special attention, for instance by way of post-discharge telephone contact or home visits from outreach services.

Somewhat surprisingly, in our study self-discharge occurred most frequent on weekday late afternoons and evenings, while during the lesser staffed night shifts larger proportions were medically discharged. However, adolescents and young adults with ethanol poisoning dominate among patients with acute poisoning at the OAEOC at night, especially during weekends [20]. During the inclusion period, these patients were eligible for a targeted follow-up program at the OAEOC [30], possibly also affecting the discharge process.

### Strengths and limitations

The information on deaths is reliable, as all included patients were traced in the National Cause of Death Register using their unique Norwegian national identity number. This is a major strength. However, our study was probably underpowered concerning factors associated with death due to the short period of observation for fatalities.

To add power to the Cox regression analysis, we chose to use death within the inclusion period or the following three months as a measure of short-time mortality, hence an observation time varying from three to fifteen months between patients. Patients with several poisonings during the inclusion period are more likely to present for the first time earlier in the inclusion period than non-repeating patients, thus having longer observation times. As self-discharge was associated with repeated poisoning, self-discharging patients might also tend to have longer observation times. This might overestimate the short-time mortality among self-discharging patients when comparing proportions between the groups. However, this ratio was in the same range as the unadjusted hazard ratio. Accordingly, we do not think that this possible bias had any impact on our results, as variation in observation time is handled by the Cox regression analysis.

For the subgroup of self-discharging patients merely disappearing during treatment, time of discharge was defined as when they were registered as missing from the clinic. Patients with acute poisoning by substances of abuse are observed at short intervals by the nursing staff at the OAEOC. When a patient is not found, this is marked in the electronic medical records. Thus, observation time is systematically somewhat overestimated for this subgroup, but on average probably by less than 15 min.

Our study included the majority of acute poisonings by substances of abuse during one year in a European capital city. However, about 200 patients with acute poisoning by substances of abuse are triaged for direct hospital treatment by the ambulance services every year, thus bypassing the OAEOC [19]. These are more severe poisonings. Furthermore, in about 700 cases per year, the patient is left on scene after treatment by the ambulance service [31]. These are mainly opioid overdoses. Consequently, the repetition rate is probably underestimated in our study, especially for patients with opioid overdoses.

The registration of previous history of severe mental illness was based on the information available then and there to the doctor treating the patient. Consequently, the prevalence is probably underestimated. Furthermore, the category of severe mental illness was not rigorously defined and encompassed a number of different diagnoses. Similarly, diagnoses of toxic agents and suicidal intention were made by the doctor treating the patient. Toxicological laboratory analyses were not done. On the other hand, the diagnoses were made in real clinical situations, and the patients were managed accordingly.

## Conclusions

Self-discharge was frequent among patients treated for acute poisoning by substances of abuse and was an independent risk factor for repeated poisoning. Short-time mortality was twice as high among self-discharging patients, though this increase was not statistically significant. Still, the increased risk of repetition marks self-discharging patients as a vulnerable group who might benefit from targeted post-discharge follow-up measures. Emergency services should give special attention to patients self-discharging during treatment for acute poisoning, for instance by way of post-discharge telephone contact or home visits from outreach services.

## Abbreviations

CI: Confidence interval
GHB: Gamma-hydroxybutyrate
OAEOC: The Oslo Accident and Emergency Outpatient Clinic
